# Identification of Specific Long Non-Coding Ribonucleic Acid Signatures and Regulatory Networks in Prostate Cancer in Fine-Needle Aspiration Biopsies

**DOI:** 10.3389/fgene.2020.00062

**Published:** 2020-02-14

**Authors:** Zehuan Li, Jianghua Zheng, Qianlin Xia, Xiaomeng He, Juan Bao, Zhanghan Chen, Hiroshi Katayama, Die Yu, Xiaoyan Zhang, Jianqing Xu, Tongyu Zhu, Jin Wang

**Affiliations:** ^1^ Scientific Research Center, Shanghai Public Health Clinical Center, Fudan University, Shanghai, China; ^2^ Department of General Surgery, Zhongshan Hospital, Fudan University, Shanghai, China; ^3^ Department of Laboratory Medicine, Zhoupu Hospital Affiliated to Shanghai University of Medicine & Health Sciences, Shanghai, China; ^4^ Department of Laboratory Medicine, Shanghai Jiao Tong University Affiliated Sixth People's Hospital, Shanghai, China; ^5^ Department of Molecular Oncology, Okayama University Graduate School of Medicine, Dentistry and Pharmaceutical Sciences, Okayama, Japan; ^6^ Department of Urology, Shanghai Key Laboratory of Organ Transplantation, Zhongshan Hospital, Fudan University, Shanghai, China

**Keywords:** prostate cancer, long non-coding ribonucleic acid, regulatory networks, fine needle aspiration biopsies, microribonucleic acid, ribonucleic acid binding proteins, biomarker

## Abstract

Prostate cancer (PCa) is one of the most common tumors in men and can be lethal, especially if left untreated. A substantial majority of PCa patients not only are diagnosed based on fine needle aspiration (FNA) biopsies, but their treatment choices are also largely driven by the pathological findings obtained with these FNA specimens. It is widely believed that lncRNAs have strong biological significance, but their specific functions and regulatory networks have not been elucidated. LncRNAs may serve as key players and regulators of PCa carcinogenesis and could be novel biomarkers of this cancer. To identify potential markers for early detection of PCa, in this study, we employed a competing endogenous RNA (ceRNA) microarray to identify differentially expressed lncRNAs (DelncRNAs) in PCa tissue and quantitative real-time PCR (qRT-PCR) analysis to validate these DelncRNAs in FNA biopsies. We demonstrated that a total of 451 lncRNAs were differentially expressed in four pairs of PCa/adjacent tissues, and upregulation of the lncRNAs RP11-33A14.1, RP11-423H2.3, and LAMTOR5-AS1 was confirmed in FNA biopsies of PCa by qRT-PCR and was consistent with the ceRNA array data. The association between the expression of the lncRNA LAMTOR5-AS1 and aggressive cancer was also investigated. Regulatory network analysis of DelncRNAs showed that the lncRNAs RP11-33A14.1 and RP11-423H2.3 targeted miR-7, miR-24-3p, and miR-30 and interacted with the RNA binding protein FUS. Knockdown of these DelncRNAs in PCa cells also demonstrated the effects of RP11-423H2.3 on miR-7/miR-24/miR-30 or LAMTOR5-AS1 on miR-942-5p/miR-542-3p *via* direct interaction. The results of these studies indicate that these three specific lncRNA signatures and regulatory networks might serve as risk prediction and diagnostic biomarkers for prostate cancer, even in biopsies obtained by FNA.

## Introduction

Prostate cancer is the second most common tumor among men worldwide, leading to the highest morbidity and mortality along with lung and bronchial cancer. In 2018, the incidence of prostate cancer (PCa) among all new cancer cases was 19%, and in the USA, ~29,000 men died from prostate cancer (Siegel et al., 2017; Siegel et al., 2018), which is usually diagnosed at a localized stage by the combination of prostate-specific antigens (PSAs), magnetic resonance imaging (MRI), digital rectal examination (DRE), and transrectal ultrasound (TRUS)-guided biopsy (Carroll et al., 2018); most panel members favor informed testing beginning at the age of 45 years. Despite these detection methods and systemic therapies, including radiation therapy, prostatectomy, androgen deprivation therapy, immunotherapy, and chemotherapy (Mohler et al., 2018), several patients are still diagnosed at a late stage of development (Siegel et al., 2018). Moreover, while PCa remains indolent in most individuals, in a minority of patients, PCa behaves aggressively. PSA, which is the most common prostatic marker, has a high specificity for prostate cancer, but its expression cannot be detected in ~5% of patients with high-grade PCa (Epstein, 1993; Van Der Toom et al., 2019) or, conversely, leads to the overdiagnosis of clinically insignificant cancer (Tan et al., 2019). Thus, biomarkers that accurately diagnose prostate cancer and, more importantly, differentiate indolent from life-threatening prostate cancer are urgently required.

Noncoding RNAs (ncRNAs) play key roles in cancer progression and could be used to develop novel biomarkers of prostate cancer (Shan et al., 2017; Xia et al., 2018). Answering the many unknown questions regarding ncRNAs' participation in prostate cancer progression, such as how ncRNAs participate in many pathological processes leading to the development of prostate cancer, how they significantly interact with proteins, and the degree of their specificity and ease of detection in tissues, serum, plasma, and urine could lead to the development of novel biomarkers of this aggressive cancer. In our previous studies, we demonstrated that four differentially expressed genes (TGBL1, HOXA7, KRT15, and TGM4) in FNA biopsies could facilitate the diagnosis of prostate cancer, which was significantly improved over PSA (Shan et al., 2017), and we found that differentially expressed circular RNAs (circRNAs) (circ_0062019 and circ_0057558) and the host gene SLC19A1 of circ_0062019 could be used as potential novel biomarkers of PCa (Xia et al., 2018). Long noncoding RNAs (lncRNAs) are currently defined as RNA transcripts longer than 200 nucleotides that do not appear to code proteins but control cell fate during development through complex mechanisms, and their dysregulation underlies some human disorders caused by chromosomal deletions and translocations (Batista and Chang, 2013). LncRNAs include several types of RNA transcripts, such as antisense, intronic, and intergenic transcripts, pseudogenes, and retrotransposons (Lee, 2012), which are more cell type-specific than protein-coding genes, and their aberrant expression has been documented in various cancers, including PCa (Hon et al., 2017; Misawa et al., 2017). LncRNAs were found to be involved in prostate carcinogenesis by mediating enhancer-promoter looping, alternative splicing, and antisense gene silencing, antagonizing transcription regulators and repressing DNA repair (Walsh et al., 2014). For example, the lncRNA SChLAP1 promotes aggressive PCa mechanistically by impairing the SWI/SNF axis-mediated regulation of their gene expression and genomic binding (Prensner et al., 2013). The lncRNA NEAT1, which is regulated by estrogen receptor alpha (ERα), drives an oncogenic cascade in PCa and is associated with therapeutic resistance (Chakravarty et al., 2014). The lncRNA HOTAIR increases the androgen receptor-mediated transcriptional program and promotes the growth of castration-resistant prostate cancer (Zhang et al., 2015). Other lncRNAs, such as lncRNA ZEB1-AS1 (Su et al., 2017) and lncRNA HOXD-AS1 (Gu et al., 2017), can also regulate cell proliferation and chemoresistance as oncogenes. However, some lncRNAs, such as lncRNA TUG1 and lncRNA CTB-89H12.4, can mediate sponge regulatory networks as tumor suppressors (Du et al., 2016). Preclinically, the interfering lncRNA MALAT1 can suppress enzalutamide-resistant PCa progression (Wang et al., 2017b). Therefore, lncRNAs play multifaceted roles in PCa and may serve as risk prediction, diagnostic, prognostic, and predictive biomarkers of PCa.

In this study, we applied a competing endogenous RNA (ceRNA) microarray to identify differentially expressed lncRNAs in PCa tissue. Through further validation of the most differentially expressed lncRNAs in prostate biopsy tissues, we found that three lncRNAs, i.e., RP11-33A14.1, RP11-423H2.3, and LAMTOR5-AS1, and their regulatory networks may serve as novel diagnostic biomarkers of PCa.

## Materials and Methods

### Cell Lines and Cell Culture

The prostate cancer cell lines 22Rv1 (ATCC No. CRL-2505), DU145 (ATCC No. HTB-81), LNCaP (ATCC No. CRL-1740), and PC3 (ATCC No. CRL-1435) were purchased from the Culture Collection of the Chinese Academy of Sciences, Shanghai, China (http://www.cellbank.org.cn/). DU145 and PC3 were cultured in MEM (Cat^#^: 41500034, Life Technologies) and F-12 (GIBCO, 21700075, Life Technologies), respectively; LNCaP and 22Rv1 were maintained in RPMI-1640 (Cat^#^: 31800022, Life Technologies) supplemented with 10% fetal bovine serum (FBS) (Thermo Fisher Scientific, Waltham, MA, US) at 37°C in 5% CO2. The human prostatic epithelial cell lines (HPEpic) were purchased from Shanghai Xinyu Biological Technology Co., Ltd. All cells were cultured according to the ATCC standard procedure.

### Prostate Tumor and Benign Prostatic Hyperplasia Tissue Samples

Four pairs of fresh prostate tumor and paracancerous tissues and 105 cases of prostate tissues on fine needle biopsies (FNA), including 48 cases of PCa tissues and 57 cases of benign prostatic hyperplasia (BPH) tissues, were acquired from Zhongshan Hospital Affiliated with Fudan University. This research was approved by the Ethics Committee of Zhongshan Hospital Affiliated with Fudan University and Shanghai Public Health Clinical Center. Written informed consent was obtained from all patients for the use of their tissue samples and clinical records. Each tissue was confirmed by a pathologist specializing in prostate cancer, and a Gleason score was provided for the risk stratification. All samples were stored at −80°C after surgical resection.

### Ribonucleic Acid Purification, Competing Endogenous Ribonucleic Acid Microarray, and Data Analysis

Total RNA was extracted and purified using TRIzol reagent (Invitrogen, Carlsbad, CA, US) and an RNeasy Mini Kit (QIAGEN, GmBH, Germany) following the manufacturer's instructions. The total RNA was quantified by a NanoDrop 2000 spectrophotometer (NanoDrop, US) and selected by limiting the 260/280 nm absorbance ratio of the samples to 1.8–2.0. The selected RNA samples were assessed by an Agilent Bioanalyzer 2100 (Agilent Technologies, Santa Clara, CA, US) to inspect the RNA integrity. Four pairs of prostate tumor and paracancerous tissues were used for the microarray assay to investigate the differentially expressed lncRNAs between the cancer tissues and paracancerous tissues (Xia et al., 2018). The total RNA was amplified and labeled by a Low Input Quick Amp WT Labeling Kit (Santa Clara, CA, US) and labeled by Cy3-labeled CTP with T7 RNA polymerase. The labeled cRNAs were purified by an RNeasy Mini Kit (QIAGEN, GmBH, Germany) and loaded onto SBC human (4*180 K) ceRNA microarrays, including 68,423 ncRNAs, 88,371 circRNAs, and 18,853 messenger RNAs (mRNAs) (Shanghai Biotech Co., Ltd., Shanghai, China). The microarray hybridization was performed following the manufacturer's standard protocols using a Gene Expression Hybridization Kit (Santa Clara, CA, US) in a hybridization oven (Santa Clara, CA, US). The hybridized slides were washed, fixed, and finally scanned to obtain images using an Agilent Microarray Scanner (Agilent Technologies, Santa Clara, CA, US). The data were extracted with Feature Extraction software 10.7 (Agilent Technologies, Santa Clara, CA, US), and the raw data were normalized by the quantile algorithm in the limma package in R. The significantly differentially expressed lncRNAs between the prostate cancer and paracancerous tissues were identified and retained by screening for fold change > 2.0 at p < 0.05. The prostate cancer microarray datasets were deposited in the Gene Expression Omnibus (GEO) database under accession number GSE140927.

### Regulatory Network Analysis of Differential Long Non-Coding Ribonucleic Acids and Microribonucleic Acids in Prostate Cancer

For an integrative analysis of prostate cancer-specific differentially expressed lncRNAs and miRNAs, we searched the GEO database for miRNA expression profiling studies related to prostate cancer. The two miRNA expression datasets were downloaded from the National Center for Biotechnology Information GEO database (GSE76260 and GSE36802). All patients' records/information were anonymized and deidentified prior to the analysis. In total, 106 prostate clinical specimens (53 cancer and 53 non-neoplastic tissues/matched benign prostate tissues) were collected from GEO to create the data downloaded from 47 patients with prostate cancer in two different platforms, including an Affymetrix Multispecies miRNA-1 Array and Illumina Human v2 MicroRNA Expression BeadChip. We applied unpaired Student's t-tests to determine the expression differences between the groups. The differential expression values are displayed as a log of the fold-change. All analyses were performed with R statistical software. We predicted the candidate genes targeted by these miRNAs based on TargetScan (Whitehead Institute for Biomedical Research, Cambridge, MA, US) (Lewis et al., 2003) or miRecords (LC Sciences, Houston, TX, US) (Xiao et al., 2009). We also applied GEO2R to determine the involvement of dysregulated miRNAs in PCa and used the microRNA.org databases and the hypergeometric method to calculate the p-values in the miRNA target analysis. Furthermore, we analyzed the potential target microRNAs (miRNAs) of the differential lncRNAs online (http://www.mircode.org). To understand the protein-lncRNA interactions of the differentially expressed lncRNAs, we constructed a lncRNA-mRNA network based on the transcripts. By analyzing the possible combination of lncRNAs and mRNAs, we predicted the target mRNAs of the differentially expressed lncRNAs (http://starbase.sysu.edu.cn/starbase2/) (Li et al., 2014) and generated a lncRNA-mRNA regulatory network map by Cytoscape3.5.1 software (Shannon et al., 2003).

### Knockdown of Differentially Expressed Long Non-Coding Ribonucleic Acids in Prostate Cancer Cells

We applied si-RP11-423H2.3 and si-LAMTOR5-AS1 to knock down the expression of RP11-423H2.3 and LAMTOR5-AS1 in the prostate PC3 and DU145 cancer cells (the target sequence of RP11-423H2.3 was AAGGACAGCTTGCCTGACT; the target sequence of LAMTOR5-AS1 was CTGGTCTACTGTCACAACA; and siRNA-GFP was the control). All siRNAs were designed and synthesized by Ribobio (Guangzhou, China). qRT-PCR was applied to validate the transfection efficiency and expression level of relevant lncRNAs and target miRNAs. The siRNA with the best transfection efficiency was selected for subsequent experiments. Prostate PC3 and DU145 cancer cells were transfected with siRNAs at a concentration of 50 nM using 5 µl of Lipofectamine 3000 (Invitrogen, CA, US) according to the manufacturer's protocol.

### Quantitative Real-Time Polymerase Chain Reaction Analysis

Total RNA was isolated from 105 clinical specimens and prostate cells using TRIzol reagent (Invitrogen, Carlsbad, CA, USA). In total, 600 ng of total RNA per sample was used for complementary DNA (cDNA) synthesis using a PrimeScript™ RT Reagent Kit with gDNA Eraser (Takara, Cat^#^: RR047A, Japan). Real-time quantitative reverse transcription PCR (qRT-PCR) was performed with SYBR Premix Ex Taq™ II (Takara, Cat^#^: RR820A, Japan) using the LightCycler 480 II Instrument (Roche Molecular Systems, Inc). We performed qRT-PCR in a total reaction volume of 10 μl, including 5 μl of 2 x SYBR Green PCR buffer, 0.4 μl of forward primer (10 μM), 0.4 μl of reverse primer (10 μM), 0.2 μl of ROX Reference Dye II, 3.5 μl of ddH_2_O, and 15 ng of cDNA. The reaction was initiated at 95°C for 1 min followed by 95°C (5 s) and 60°C (30 s) for 40 cycles. The expression of the lncRNAs was normalized to the level of 18S. The specific primers of the lncRNAs, miRNAs, and 18S are presented in **Table S1**. The data were collected and analyzed using the 2^−ΔΔCt^ method.

### Immunoblots

Prostate PC3 and DU145 cancer cells were transfected with si-RP11-423H2.3, si-LAMTOR5-AS1, or siRNA-GFP (si-Control) using Lipofectamine 3000 (Invitrogen, CA, US) according to the manufacturer's protocol. After 72 h, protein samples were lysed in radioimmunoprecipitation assay (RIPA) buffer supplemented with protease inhibitors. Thirty micrograms of total protein were loaded per lane separated on a 10% sodium dodecyl sulfate (SDS)-polyacrylamide gel by electrophoresis, and proteins transferred onto nitrocellulose membranes. The membranes were blocked with 5% milk in phosphate buffered saline with tween 20 (PBST) and then incubated with a rabbit anti-UPF1 (Cat#: D161327, BBI Solutions) or rabbit anti-FUS (Cat#: D223360, BBI Solutions), or β-actin (N-21) rabbit polyclonal antibody (Cat#: sc-130656, Santa Cruz Biotechnology, Inc) at 4°C overnight. After washing with PBST, the blots were treated with a horseradish peroxidase (HRP) conjugated anti-rabbit IgG. Detection of blots was performed using Meilunbio^®^ fg super sensitive ECL luminescence reagent (Dalian Meilun Biotechnology Co., Ltd.) (Zhang et al., 2019).

### Statistical Analyses

We collected clinical data from 105 prostate tissues, and a Student's t-test was used to analyze the differences in lncRNA expression between the prostate cancer group and BPH group. A Pearson correlation analysis was used to investigate the relationship between the differential lncRNAs and clinical parameters. The results were regarded as statistically significant at p < 0.05. All graphs were generated using GraphPad Prism 7.0 software (GraphPad Software Inc., La Jolla, CA, USA). The statistical analysis was performed using SPSS 22.0 (IBM-SPSS Inc., Chicago, IL, USA). Receiver operating characteristic (ROC) curves were applied to evaluate the clinical diagnostic value of the differential lncRNAs and the combination of PSA and lncRNAs.

## Results

### Differential Profiling of Long Non-Coding Ribonucleic Acids in Prostate Cancer

**Figure 1:**
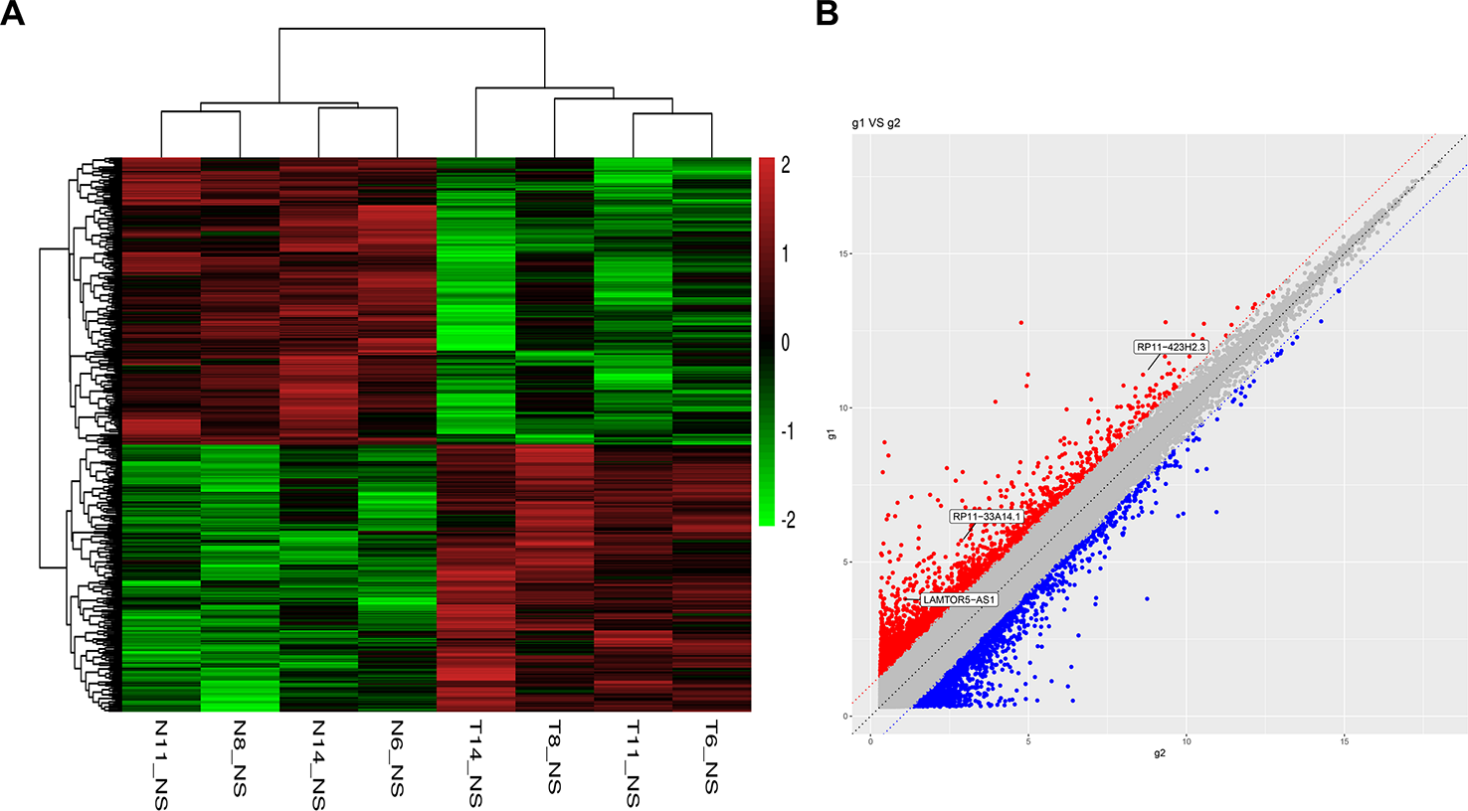
Heatmap and scatter plots of differential long non-coding RNAs (lncRNAs) in prostate tumor tissues and normal tissues. **(A)** Heatmap of differential lncRNAs; **(B)** scatter plots of differential lncRNAs.

To identify potential biomarkers of PCa, we first performed ceRNA microarray profiling of PCa patients and detected many transcripts in the PCa and adjacent normal tissues. We collected four pairs of tumor/adjacent normal tissue paraffin specimens and applied a ceRNA microarray to detect the transcripts in the PCa and adjacent normal tissues (Xia et al., 2018). A heatmap (**Figure 1A**) and scatter plots (**Figure 1B**) of the differential lncRNAs between the PCa tissues and normal tissues are shown in **Figure 1**. The heatmap indicates that 451 lncRNAs (**Figure 1A**) were differentially expressed with a fold change > 2.0 at p < 0.05. Among these lncRNAs, 217 lncRNAs were upregulated, and 234 lncRNAs were downregulated, in four pairs of PCa/adjacent tissues (**Table 1**). Among the differentially expressed lncRNAs, the most upregulated lncRNA is LINC00675, and the most downregulated lncRNA is RP11-864N7.4.

**Table 1:** Top 10 of the differentially expressed long non-coding ribonucleic acids (lncRNA) in prostate cancer (PCa) (cancer/paracancerous tissue).

Accession	Gene symbol	Relation	Fold change	P values	FDR
NR_036581	LINC00675	intergenic	7.58	0.045	0.59
ENST00000439575	RP11-118K6.2	intergenic	7.41	0.023	0.55
ENST00000609245	LAMTOR5-AS1	intergenic	7.30	0.001	0.47
ENST00000414475	RP11-33A14.1	intergenic	6.96	0.049	0.59
lnc-NTM-4:1	—	intergenic	6.27	0.030	0.55
ENST00000503263	RP11-423H2.3	intergenic	5.46	0.024	0.55
ENST00000366189	RP11-423H2.3	intergenic	5.13	0.047	0.59
lnc-KAZALD1-1:1	—	intronic_sense	5.11	0.044	0.59
ENST00000605909	RP11-16D22.2	intergenic	4.71	0.012	0.51
ENST00000623288	RP11-423H2.4	intergenic	4.51	0.032	0.56
ENST00000371162	MIR4435-1HG	intergenic	−3.76	0.018	0.53
lnc-PRICKLE2-6:1	—	exonic_sense	−3.79	0.041	0.58
lnc-AC079135.1.1-8:1	—	exonic_sense	−3.92	0.032	0.56
lnc-ZDHHC13-5:1	—	exonic_sense	−4.19	0.027	0.55
ENST00000451884	MIR4435-1HG	intergenic	−4.19	0.003	0.47
lnc-JPH2-1:1	—	exonic_sense	−4.54	0.009	0.50
lnc-C15orf54-4:2	—	exonic_sense	−4.59	0.010	0.50
lnc-TPD52L3-1:1	—	exonic_sense	−5.31	0.013	0.51
NONHSAT018709	—	exonic_sense	−6.45	0.015	0.51
ENST00000624759	RP11-864N7.4	intronic_sense	−15.56	0.025	0.55

### Validation of Key Differentially Expressed Long Non-Coding Ribonucleic Acids (RP11-33A14.1, RP11-423H2.3, and LAMTOR5-AS1) Using Fine Needle Aspiration Samples

**Figure 2:**
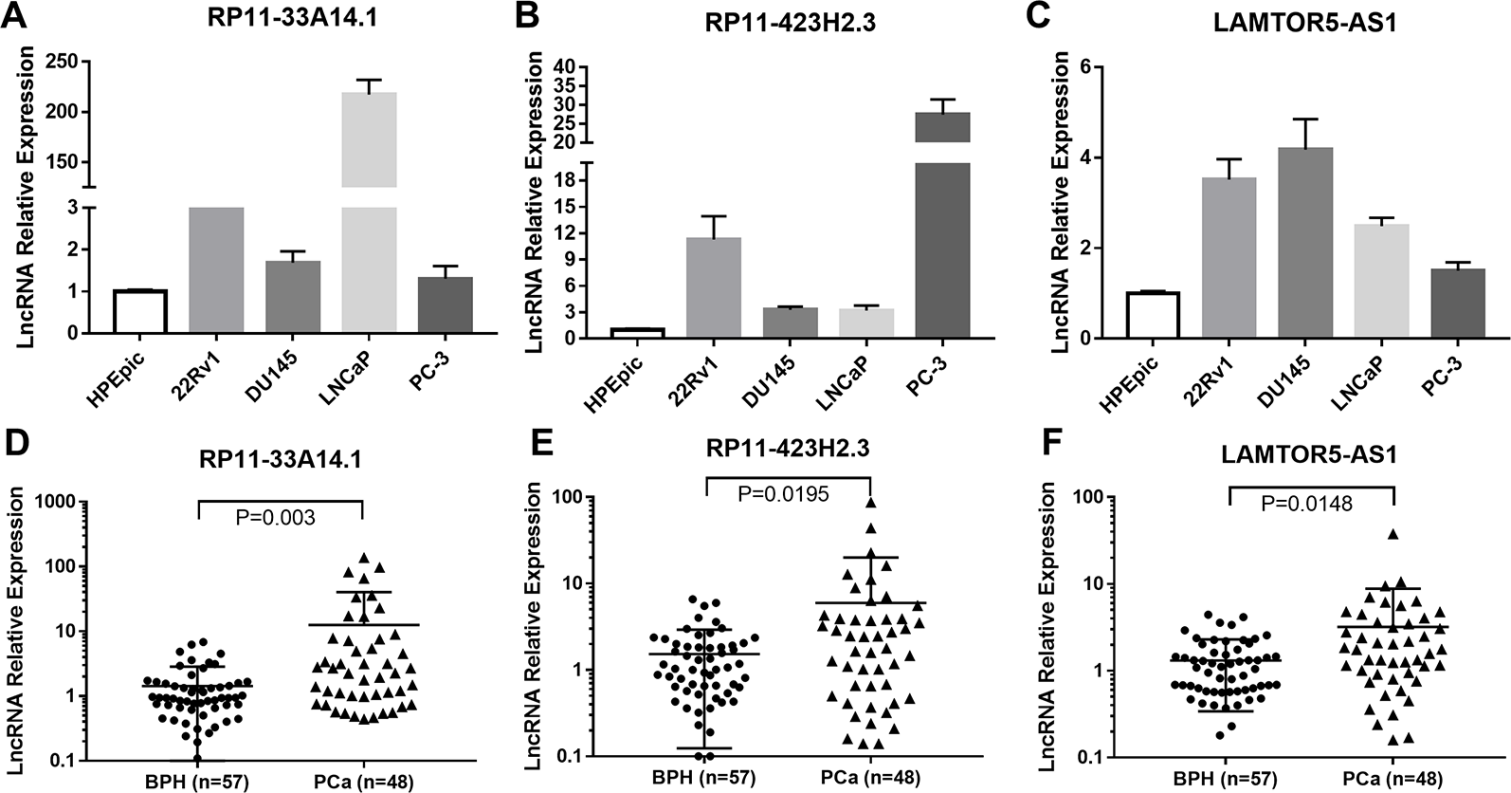
Quantitative real-time (qRT)-PCR analysis of the gene expression levels of lncRNAs (RP11-33A14.1, RP11-423H2.3, and LAMTOR5-AS1) in prostate cells and tumor tissue fine needle aspiration (FNA) samples. RP11-33A14.1 **(A, D)**, RP11-423H2.3 **(B, E)**, and LAMTOR5-AS1 **(C, F)** in prostate cells **(A–C)**, and tumor tissue samples compared to benign prostatic hyperplasia (BPH) tissue samples **(D–F)**.

We further carried out a qRT-PCR analysis of the related differential lncRNAs, including RP11-33A14.1, RP11-423H2.3, LAMTOR5-AS1, LINC00675, RP11-118K6.2, and RP11-423H2.3, in the normal prostate cell line HPEpic, PCa cells (22Rv1, DU145, LNCaP, and PC3 cells), and 105 FNA prostate tissues (48 PCa tissues and 57 BPH tissues) (**Figure S1**). The results revealed that the lncRNAs RP11-33A14.1 (**Figure 2A**), RP11-423H2.3 (**Figure 2B**), and LAMTOR5-AS1 (**Figure 2C**) were upregulated in the four PCa cells. We further validated these lncRNAs in 48 PCa tissues and 57 BPH tissues. The results showed that in the PCa tissues, the lncRNAs RP11-33A14.1, RP11-423H2.3, and LAMTOR5-AS1 were upregulated by 11.12 ± 3.66-fold (**Figure 2D**), 4.44 ± 1.87-fold (**Figure 2E**), and 1.89 ± 0.76-fold (**Figure 2F**), respectively (p < 0.05), further confirming the results of our ceRNA microarray.

### Differentially Expressed Long Non-Coding Ribonucleic Acids as Novel Biomarkers of Prostate Cancer Associated With Prostate-Specific Antigens Levels and the Progression of Prostate Cancer

**Figure 3:**
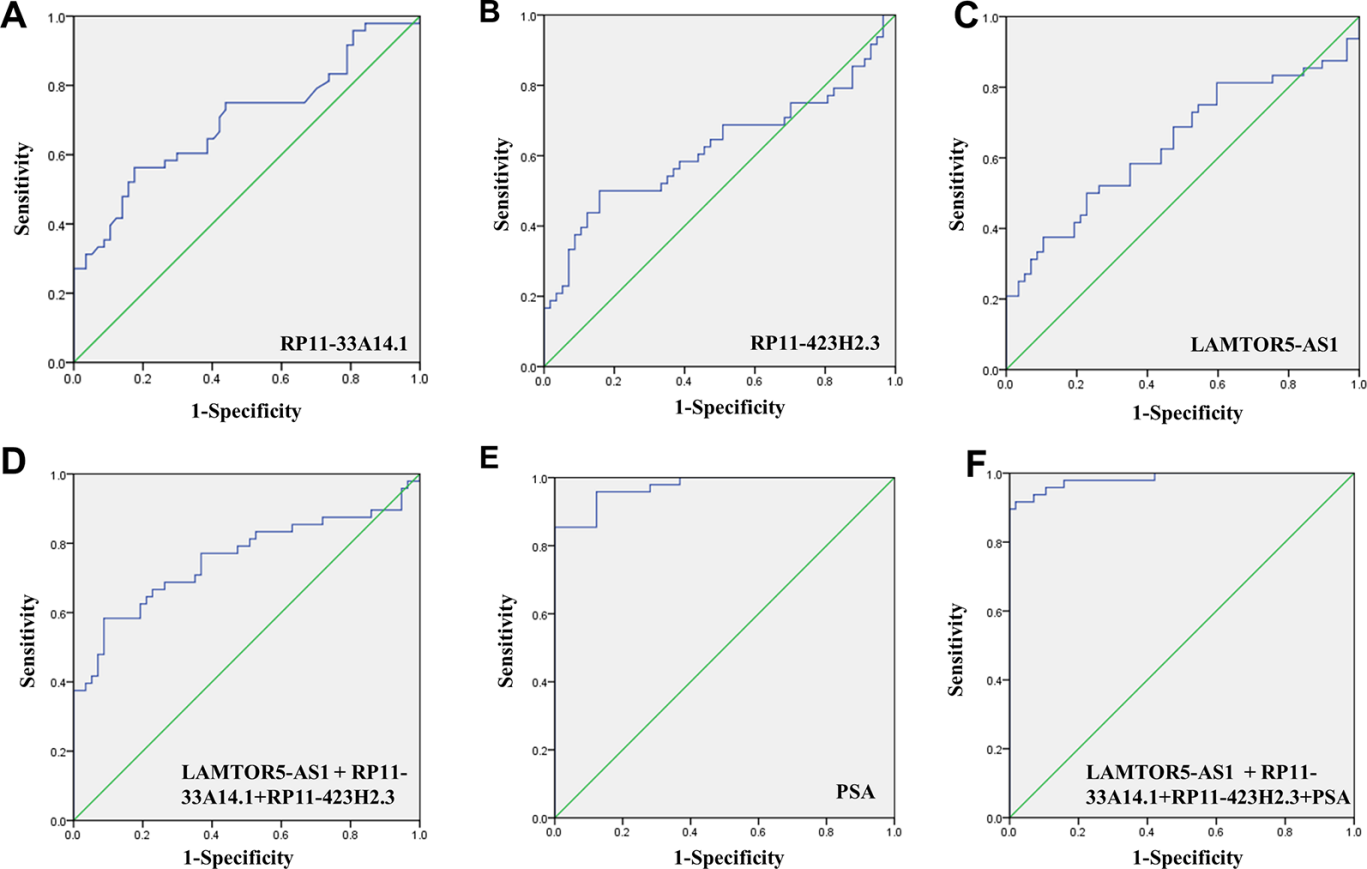
ROC curve showing expression levels of differentially expressed long non-coding RNAs (DelncRNAs). LncRNA RP11-33A14.1 **(A)**, RP11-423H2.3 **(B)**, and LAMTOR5-AS1 **(C)** in prostate cancer (PCa) patients and benign prostatic hyperplasia (BPH) controls; the three lncRNAs combination **(D)**; prostate-specific antigen (PSA) only **(E)**; and the three lncRNAs and PSA combination **(F)**. The receiver operating characteristic (ROC) curves were analyzed using univariate (log-rank) analysis.

We assessed the diagnostic effectiveness of the differential lncRNAs in differentiating between PCa and BPH tissues by an ROC curve (**Figure 3**). The areas under the curve (AUCs) of lncRNAs RP11-33A14.1, RP11-423H2.3, and LAMTOR5-AS1 were 0.697, 0.620, and 0.641, respectively (**Figure 3** and **Table 2**). When the three differential lncRNAs were combined, the AUC was 0.754 (**Figure 3D**). We further analyzed the PSA level using the results of the 3 differential lncRNAs, and the AUC was 0.984. The sensitivity was 97.9%, and the specificity was 84.2% (**Figure 3E**). To clarify the characteristics of these differential lncRNAs in PCa, we applied a Pearson correlation analysis to analyze the correlation between these lncRNAs and the corresponding clinical parameters. As shown in **Table 3**, the lncRNA LAMPOR5-AS1 is positively correlated with the PSA level of the patients (p < 0.001). A combined Gleason score of 6 or 7 indicates that PCa is likely to grow but may not spread quickly. A score of 8–10 is suggestive of aggressive prostate cancer that is potentially lethal [24]. In this study, we investigated the association between the expression of lncRNA LAMTOR5-AS1 and aggressive cancer (Gleason score 8–10, p < 0.05) (**Table 4**) and found that lncRNA LAMTOR5-AS1 expression was higher in the less aggressive PCa (Gleason score 6–7; GS6-7) than in the aggressive PCa (Gleason score 8–10; GS8-10), yet its expression in GS8-10 was higher than in non-cancer tissues (*p* = 0.023) (**Figure S2**), which indicated that LAMTOR5-AS1 might be useful in the early diagnosis of PCa.

**Table 2:** ROC analysis of the diagnostic efficiency of differential long non-coding ribonucleic acids (lncRNAs) (RP11-33A14.1, RP11-423H2.3, and LAMTOR5-AS1) and serum prostate-specific antigen (PSA) in prostate cancer (PCa) patients and benign prostatic hyperplasia (BPH) controls.

Biomarker	Sensitivity (%)	Specificity (%)	AUC (95% CI)	P-value
RP11-33A14.1	60.4	70.2	0.697 (0.506–0.734)	0.001
RP11-423H2.3	56.2	61.4	0.620 (0.506–0.734)	0.035
LAMTOR5-AS1	58.3	64.9	0.641 (0.531–0.751)	0.013
LAMTOR5-AS1 + RP11-33A14.1+RP11-423H2.3	77.1	63.2	0.754 (0.655–0.854)	<0.001
PSA	95.8	84.2	0.974 (0.946–0.998)	<0.001
LAMTOR5-AS1 + RP11-33A14.1+RP11-423H2.3 + PSA	97.9	84.2	0.984 (0.964–1.004)	<0.001

**Table 3:** Association between the differential long non-coding ribonucleic acids (lncRNAs) and clinical parameter in prostate cancer (PCa) patients.

Clinical parameter	RP11-33A14.1, r (P)*	RP11-423H2.3, r (P)*	LAMTOR5-AS1, r (P)*
Age	0.165 (0.094)	0.324 (0.001)	0.258 (0.008)
PSA	0.025 (0.799)	0.347 (0.000)	**0.803 (0.000)**
Cholesterol (TC)	−0.158 (0.281)	−0.196 (0.370)	−0.299 (0.166)
Triglyceride (TG)	−0.161 (0.463)	0.021 (0.924)	−0.215 (0.324)
Fasting plasma glucose (FPG)	0.003 (0.990)	0.455 (0.077)	−0.179 (0.506)
Gleason score	0.020 (0.941)	−0.247 (0.091)	0.243 (0.096)

*Bold values denote statistical significance at the p < 0.05 level.

**Table 4:** Association between the differential long non-coding ribonucleic acids (lncRNAs) and aggressive prostate cancer (PCa).

Histologic diagnosis	lncRNA (mean ± SD)		
	RP11-33A14.1	RP11-423H2.3	LAMTOR5-AS1
Aggressive cancer (Gleason score 8–10)	1.19 ± 1.48	3.58 ± 6.23	1.92 ± 2.10
Less aggressive cancer (Gleason score 6–7)	1.55 ± 1.46	4.61 ± 8.12	3.42 ± 3.01
p-value	0.414	0.653	**0.038**

*Bold values denote statistical significance at the *p* < 0.05 level.

### Regulatory Network Analysis of Differentially Expressed Long Non-Coding Ribonucleic Acids, Their Target Microribonucleic Acids, and Their Interaction With Ribonucleic Acids Binding Protein in Prostate Cancer

**Figure 4:**
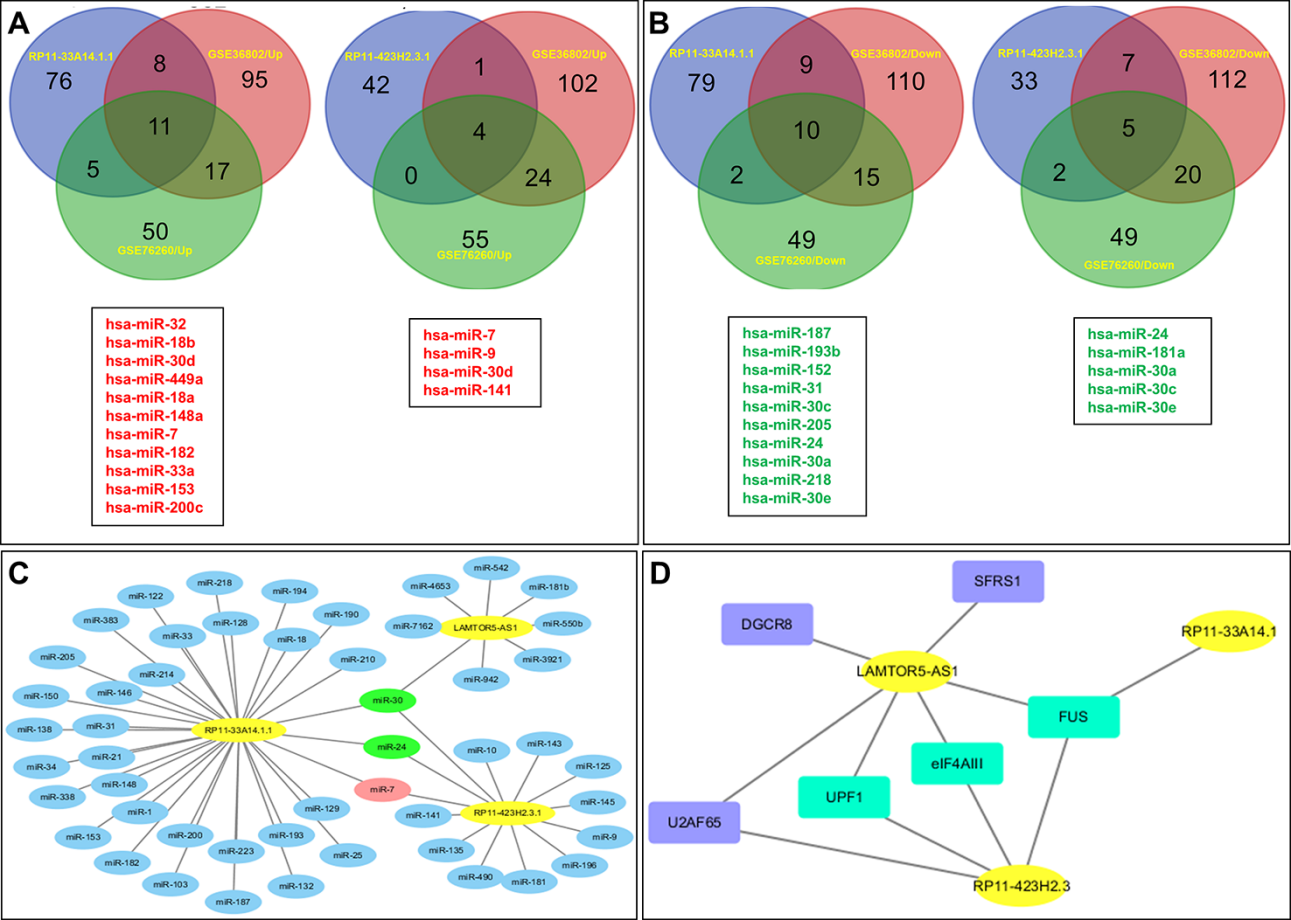
Targeted microRNAs (miRNAs) of differentially expressed long non-coding RNAs (DelncRNAs) in prostate cancer (PCa) and their regulatory network analysis. The Venn diagram demonstrates that the dysregulated miRNAs in PCa from the expression profiles of GSE76260 and GSE36802 in the GEO databases are the targeted miRNAs of DelncRNAs in PCa **(A, B)**, with upregulation in PCa **(A)** and downregulation in PCa **(B)**; regulatory network analysis of differential lncRNAs, their targeted miRNAs **(C)**; lncRNA RP11-423H2.3 and LAMTOR5-AS1 shared common RNA-binding proteins **(D)**.

Subsequently, we predicted the miRNAs likely to be targeted by these three lncRNAs. In total, 100 miRNAs with binding sites for lncRNA RP11-33A14.1 and 47 miRNAs with binding sites for lncRNA RP11-423H2.3 were selected for subsequent analysis (**Figures 4A, B**). We also analyzed the miRNA expression profiles of GSE76260 and GSE36802 from the GEO databases. The microarray dataset GSE76260 included 32 prostate cancer and 32 non-neoplastic tissue samples; GSE36802 included 21 pairs of prostate cancer samples and matched benign prostate tissues. We identified 53 miRNAs that were differentially expressed between the prostate cancer tumor tissue and the normal controls. We found that compared with the normal controls, 28 miRNAs were upregulated (**Figure 4A**), and 25 miRNAs were repressed in the prostate cancer tissue samples (**Figure 4B**) in the two GEO datasets. Taken together, these results indicate that miR-7 predicted from lncRNAs RP11-33A14.1 and RP11-423H2.3 was upregulated in the prostate cancer tumor tissue in the two GEO datasets (**Figure 4A**). In contrast, two miRNAs (miR-24 and miR-30c) predicted from the two lncRNAs were repressed in the prostate cancer tumor tissue in the two GEO datasets (**Figure 4B**). Furthermore, we found that lncRNAs RP11-33A14.1 and RP11-423H2.3 both target miR-7, miR-24-3p, and miR-30 (**Figure 4C**). However, we only obtained two predicted miRNAs (miR-542-3p and miR-30c) for LAMTOR5-AS1 if we combined these two GEO datasets and utilized the miRDB database to identify target miRNAs. Next, we applied three reference datasets, DIANA-TarBase (http://www.microrna.gr/tarbase) (Karagkouni et al., 2018), lncRNASNP2 (http://bioinfo.life.hust.edu.cn/lncRNASNP/#!/mirna/), and miRDB (http://www.mirdb.org/), to predict the targeted miRNAs of LAMTOR5-AS1 and overlapped the three predicted results. Furthermore, we selected the top miRNAs (miR-550b-3p, miR-942-5p, miR-542-3p, miR-7162-3p, miR-4653, miR-3921, and miR-181b-3p) (**Table S3**) with the highest context scores (score > 70 in two predicted datasets) to establish a lncRNA-miRNA network for LAMTOR5-AS1 (**Figure 4C**). Finally, we analyzed the regulatory networks of lncRNAs RP11-33A14.1, RP11-423H2.3, and LAMTOR5-AS1 and predicted their potential RNA binding proteins (RBPs) using the starBase database. We found that lncRNAs RP11-423H2.3 and LAMTOR5-AS1 shared common RBPs, including eIF4AIII, U2AF65, and UPF1. More intriguingly, lncRNAs RP11-33A14.1, RP11-423H2.3, and LAMTOR5-AS1 interact with the same RBP FUS (**Figure 4D**).

## Discussion

Prostate cancer is one of the most common cancers in men and ranges from low risk states amenable to active surveillance to high-risk states that can be lethal, especially if left untreated (Eskra et al., 2019). Although the diagnosis cornerstone of PCa has been prostate-specific antigen levels and numerous biomarkers have been introduced over the past decade, there is still a critical need for the development of relatively noninvasive and clinically useful methods for the screening, detection, prognosis, disease monitoring, and prediction of treatment efficacy of PCa.

Noncoding RNAs (ncRNAs) are typically classified into small and lncRNAs based on their size ranges of <200 or >200 nucleotides, and these RNAs are actively transcribed to a versatile group of RNA transcripts without protein-coding potential (over 80% of the genome) (Kapranov et al., 2007; Djebali et al., 2012). The dysregulation of lncRNAs has been implicated in the development and progression of a variety of cancers (Das et al., 2019). However, notably few lncRNAs have been functionally characterized and experimentally validated in PCa. In this study, the lncRNAs RP11-33A14.1, RP11-423H2.3, and LAMTOR5-AS1 were found to be upregulated in FNA biopsies of PCa. Several members of the lncRNA RP11 family are related to malignancies, including glioblastoma, renal cell carcinoma, and colorectal cancer. The lncRNA RP11-838N2.4 enhances the cytotoxic effects of temozolomide by inhibiting the functions of miR-10a in glioblastoma cell lines (Liu et al., 2016). The lncRNA RP11-436H11.5 functions as a ceRNA to upregulate BCL-W expression by sponging miR-335-5p, thereby promoting proliferation and invasion in renal cell carcinoma (Wang et al., 2017a). The downregulation of long noncoding RNA RP11-708H21.4 is associated with a poor prognosis in colorectal cancer and promotes tumorigenesis by regulating the AKT/mTOR pathway (Sun et al., 2017). RP11-380D23.2 drives the distal-proximal patterning of the lung by regulating PITX2 expression (Banerjee et al., 2018). The lncRNA LAMTOR5-AS1, which is known as late endosomal/lysosomal adaptor-2C MAPK and MTOR activator 5 (LAMTOR5) antisense RNA 1, was first shown to be associated with PCa in this report. Subsequently, we assessed the diagnostic effectiveness of differential lncRNAs in differentiating between PCa and BPH tissues. When the PSA level was combined with the three differential lncRNAs, the AUC was 0.984, the sensitivity was 97.9% and the specificity was 84.2%, which are better than the values obtained using PSA only. We previously demonstrated that different levels of two circRNAs (circ_0057558 and circ_0062019) and four genes DEGs (ITGBL1, TGM4, KRT15, and HOXA7) could help to distinguish PCa patients from non-PCa patients (Shan et al., 2017; Xia et al., 2018); thus, we proposed that combining these biomarkers might improve the diagnostic efficiency of PCa. We demonstrated that when the expression of two circRNAs (circ_0057558 and circ_0062019) or 4 differentially expressed genes (DEGs) (ITGBL1, TGM4, KRT15, and HOXA7) were considered along with the three differentially expressed lncRNAs (DelncRNAs), the AUC was 0.935 (**Figure S3A**) and 0.968 (**Figure S3B**), the sensitivity was 85.0% and 93.8%, and the specificity was 89.2 and 92.7%, respectively. We also attempted to include only one gene (ITGBL1) and one circRNA (circ_0062019), which were the best biomarkers for the diagnosis of PCa in our previous publications, and found that when the expression of ITGBL1 and circ_0062019 was considered along with the three DelncRNAs, the AUC was 0.957 (**Figure S3C**), the sensitivity was 93.3%, and the specificity was 92.3% (**Table S2**), which were significantly improved compared to three lncRNAs. We also demonstrated that the lncRNA LAMPOR5-AS1 is positively correlated with the PSA level in patients and is more closely related to less aggressive PCa than to aggressive PCa, indicating that LAMTOR5-AS1 may be useful in the early diagnosis of PCa and that these differentially expressed lncRNAs might be novel biomarkers of PCa.

**Figure 5:**
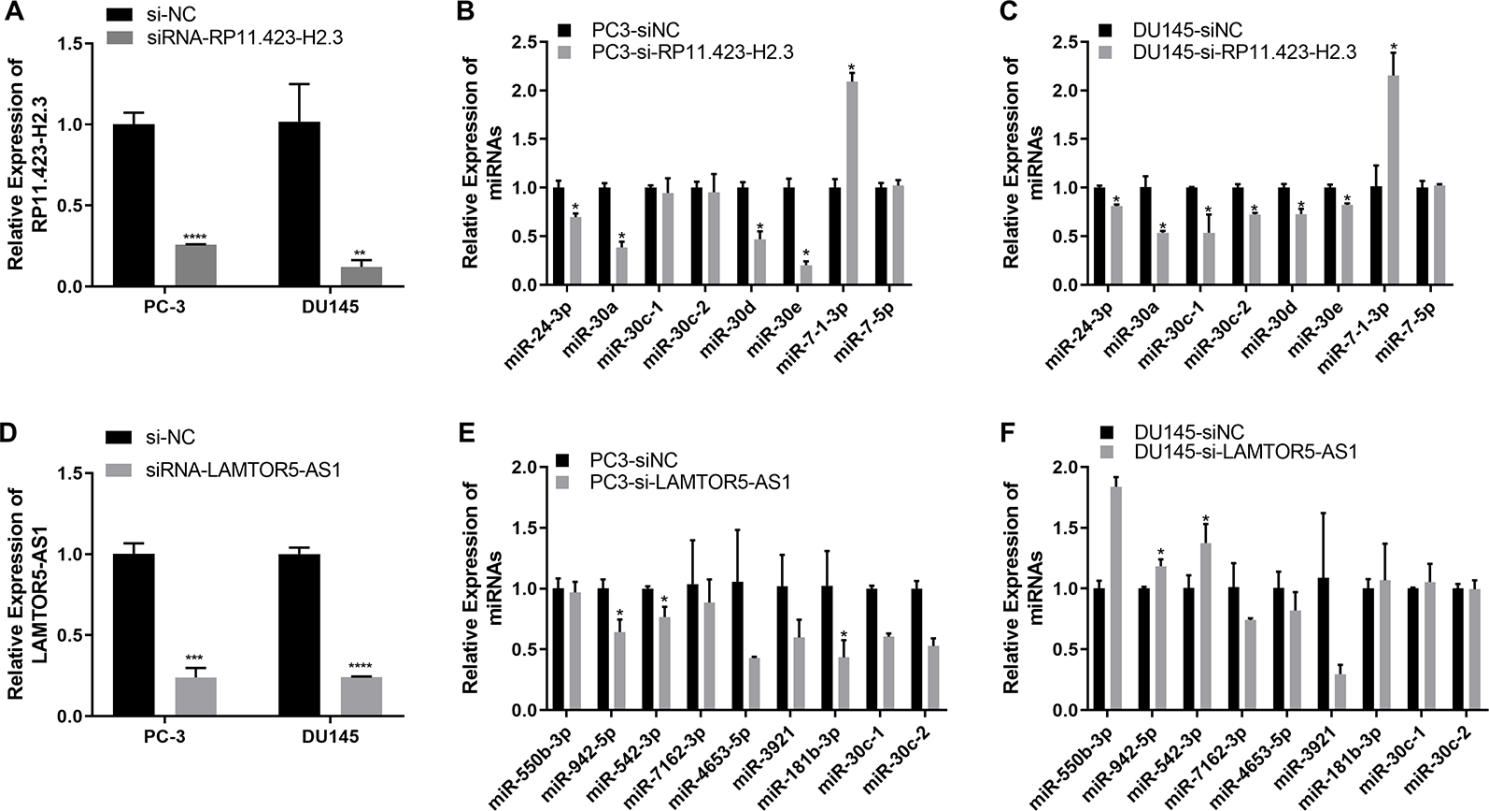
Quantitative real-time (qRT-PCR) analysis of the gene expression levels of long non-coding RNAs (lncRNAs) and target microRNAs (miRNAs) in prostate cancer (PCa) cells with knockdown of RP11-423H2.3 or LAMTOR5-AS1. Knockdown of RP11-423H2.3 **(A–C)** and LAMTOR5-AS1 **(D–F)** in prostate cells; the expression levels of target miRNAs in PC3 cells **(B, E)** and DU145 cells **(C, F)**. *p < 0.05; **p < 0.01; ***p < 0.001.

We further performed a regulatory network analysis of the differentially expressed lncRNAs and predicted that miR-7, miR-24, and miR-30 were target miRNAs of lncRNAs RP11-33A14.1 and RP11-423H2.3. Among these miRNAs, two miRNAs (miR-7 and 30d) were upregulated (**Figure 4A**), but four miRNAs (miR-24, miR-30a, miR-30c, and miR-30e) were repressed in the prostate cancer tumor tissue (**Figure 4B**) in the two GEO datasets. To determine if possible mechanisms of action that target miRNA expression were affected by these DelncRNAs, we knocked down RP11-423H2.3 and LAMTOR5-AS1 in PCa cells. Our results revealed that knockdown of RP11-423H2.3 reduced the expression levels of miR-24-3p, miR-30a, miR-30d, and miR-30e and upregulated miR-7-1-3p in both PC3 and DU145 cells (**Figures 5A–C**). We also found that when LAMTOR5-AS1 was knocked down (**Figure 5D**), miR-942-5p, and miR-542-3p were repressed in PC3 cells (**Figure 5E**) but upregulated in DU145 cells (**Figure 5F**). In keeping with the ceRNA regulatory mechanism, lncRNAs can function as molecular decoys or sponges of microRNAs (Salmena et al., 2011), which might cause increased expression of miR-7-1-3p following knockdown of RP11-423H2.3. On other hand, some lncRNAs could also be processed to generate miRNAs or activate miRNA expression (Yoon et al., 2014), so that several miRNAs were deregulated after knockdown of RP11-423H2.3 or LAMTOR5-AS1, which supported the effects of RP11-423H2.3 on miR-7/miR-24/miR-30 or LAMTOR5-AS1 on miR-942-5p/miR-542-3p *via* direct interaction. miR-7 can inhibit the stemness of prostate cancer stem-like cells and tumorigenesis by repressing the KLF4/PI3K/Akt/p21 pathway (Chang et al., 2015). miR-24 serves as a tumor suppressor role in PCa and was repressed in prostate cancer cell lines and tumor tissue, which was correlated with high PSA serum levels and related to prostate cancer progression (Lynch et al., 2016). miR-30 was also downregulated in prostate cancer cells compared to that in the prostate immortalized normal epithelial-derived cell line RWPE-1, which may be associated with tumor suppressor functions in prostate cancer (Kao et al., 2014), and miR-30 has been identified as a direct regulator of androgen receptor signaling in prostate cancer by complementary functional microRNA library screening (Kumar et al., 2016). miR-30a-5p and miR-30b were not only found to be lower in PCa tumors than in benign tissues but significantly increased when VCaP and PC3 cells were treated with saracatinib and PP2. However, miR-30c was different (Kao et al., 2014). miR-30b-3p and miR-30d-5p can be direct regulators of androgen receptor signaling in prostate cancer, and inhibition of miR-30b-3p and miR-30d-5p can increase androgen receptor (AR) expression and promote androgen-independent cell growth (Kumar et al., 2016). Finally, we determined that the lncRNAs RP11-423H2.3 and LAMTOR5-AS1 shared common RBPs, including eIF4AIII, U2AF65, and UPF1. Some lncRNAs can recruit regulatory compounds and affect gene expression by interacting with RBPs (Jia et al., 2017). The lncRNA MEG3 interacts with the RBP polypyrimidine tract-binding protein 1 (PTBP1) and induces cholestatic liver injury (Zhang et al., 2017). LncRNAs might affect the expression level of neighboring genes by a cis-regulated function. We found that all three lncRNAs, i.e., RP11-33A14.1, RP11-423H2.3, and LAMTOR5-AS1, interacted with FUS, while the loss of FUS expression may contribute to cancer progression (Brooke et al., 2011). The DNA and RNA helicase UPF1 played key roles in nonsense mediated RNA decay (NMD) that could selectively degrade aberrant RNA transcripts (Azzalin and Lingner, 2006). FUS was a multifunctional protein and participated in many RNA metabolism pathways, and mutant FUS suppressed protein biosynthesis and disrupted NMD regulation (Kamelgarn et al., 2018). FUS expression was also inversely correlated with Gleason grade of prostate cancer (Ghanbarpanah et al., 2018). We demonstrated that deregulation of FUS and UPF1 was in both PC3 and DU145 cells following knockdown of RP11-423H2.3 or LAMTOR5-AS1 (**Figure S4**). which implied that RBP FUS and UPF1 with lncRNA RP11-423H2.3 or LAMTOR5-AS1 interactions might affect prostate cancer progression. Deregulation of the RNA-binding protein fused in sarcoma/translocated in liposarcoma (FUS/TLS) in breast cancer cells by interacting with the lncRNA nuclear paraspeckle assembly transcript 1 (NEAT1) and miR-548ar could induce cell apoptosis (Wang et al., 2016). As FUS is a member of the TET protein family, this protein was found to be inversely regulated by miR-141 in human neuroblastoma (Wang et al., 2016) and can be activated by lncRNA XIST, which also served as a ceRNA in cervical cancer progression while competitively binding with miR-200a (Zhu et al., 2018). FUS promoted conditions that favored cell-cycle arrest by reducing proliferator factors and was a key link between androgen receptor signaling and the progression of the cell cycle in prostate cancer (Brooke et al., 2011; Ghanbarpanah et al., 2018).

## Conclusions

While we continue to search for smarter and more reliable, precise, and cost-effective screening methods, we continue to advocate shared decision-making in prostate cancer screening to serve our patients' best interests. The differentially expressed lncRNAs and their specific regulatory networks may serve as potential biomarkers for the clinical diagnosis and treatment of PCa, which could guide decisions regarding whom to biopsy and whom to re-biopsy after an initial negative biopsy with continued suspicion of PCa and might support an individual oncological approach in the future.

## Data Availability Statement

The prostate cancer microarray datasets were deposited in the Gene Expression Omnibus (GEO) database under accession number GSE140927.

## Ethics Statement

The studies involving human participants were reviewed and approved by the Ethics Committee of Zhongshan Hospital Affiliated with Fudan University and Shanghai Public Health Clinical Center. Written informed consent was obtained from all patients for the use of their tissue samples and clinical records.

## Author Contributions

JW and JZ planned overall concepts and designed the experiments. ZL, QX, XH, ZC, and DY performed the experiments. QX, ZL, HK and JW interpreted the data. XZ, TZ, JB and JX supported the study. ZL participated in drafting the manuscript. JW wrote and revised the manuscript.

## Conflict of Interest

The authors declare that the research was conducted in the absence of any commercial or financial relationships that could be construed as a potential conflict of interest.

## Abbreviations

AUC, area under the curve; BPH, benign prostatic hyperplasia; ceRNA, competing endogenous RNA; DEGs, differentially expressed genes; DelncRNAs, differentially expressed lncRNAs; DRE, digital rectal examination; ERα, estrogen receptor alpha; FNA, Fine-Needle Aspiration; FPG, fasting plasma glucose; FUS/TLS, fused in sarcoma/translocated in liposarcoma; GEO, gene expression omnibus; lncRNAs, long noncoding RNAs; MRI, magnetic resonance imaging; ncRNAs, noncoding RNAs; NEAT1, nuclear paraspeckle assembly transcript 1; NMD, nonsense mediated RNA decay; PCa, prostate cancer; PSA, prostate specific antigen; PTBP1, polypyrimidine tract-binding protein 1; qRT-PCR, quantitative real-time polymerase chain reaction; ROC, receiver operating characteristic; RBPs, RNA binding proteins; TC, total cholesterol; TG, total triglyceride; TRUS, transrectal ultrasound.
