# Supplementary material for: Identification of Specific Long Non-Coding Ribonucleic Acid Signatures and Regulatory Networks in Prostate Cancer in Fine-Needle Aspiration Biopsies
Source: Front Genet. 2020 Feb 14;11:62. doi: 10.3389/fgene.2020.00062 (PMC7034103; doi:10.3389/fgene.2020.00062)

Supplemental materials

Identification of specific lncRNA signatures and regulatory networks in prostate cancer in fine-needle aspiration biopsies

Zehuan Li [1, 2, #], Jianghua Zheng [3, #], Qianlin Xia[4, #], Xiaomeng He[1, #], Juan Bao[1], Zhanghan Chen[2], Hiroshi Katayama[5], Die Yu[1], Xiaoyan Zhang[1], Jianqing Xu[1], Tongyu Zhu[1,6], Jin Wang[1, *]

[1]Scientific Research Center, Shanghai Public Health Clinical Center, 2901 Caolang Road, Jinshan District, Shanghai, China; [2]Department of General Surgery, Zhongshan Hospital, Fudan University, Shanghai, China; [3]Department of Laboratory Medicine, Zhoupu Hospital Affiliated to Shanghai University of Medicine & Health Sciences, Shanghai, China;[4]Department of Laboratory Medicine, Shanghai Jiao Tong University Affiliated Sixth People's Hospital, Shanghai, China; [5]Department of Molecular Oncology, Okayama University Graduate School of Medicine, Dentistry and Pharmaceutical Sciences , Okayama , Japan; [6]Department of Urology, Shanghai Key laboratory of Organ Transplantation, Zhongshan Hospital, Fudan University, 180 Fenglin Road, Shanghai, 200032, China.

[#] These authors contributed equally to this work

[*]Corresponding Author:

Jin Wang, Ph.D.

Scientific Research Center, Shanghai Public Health Clinical Center,

2901 Caolang Road, Jinshan District, Shanghai 201508, China;

Ph: 86-21-37990333-7336;

Fax: 86-21-57247094

Email: wjincityu@yahoo.com

1.1.Table S1. Sequences of primers for real-time qPCR.

Gene	Primer	Sequence (5'-3')
RP11-423H2.3	Forward	ACAGGGTATCACCAGACTCCT
	Reverse	CTACTGCGCCCGTAAGAACC
LAMTOR5-AS1	Forward	CAGAGAAGAGAATGTCATGGAGA
	Reverse	CAAGGGGAATGTGGGAGTC
RP11-33A14.1	Forward	GCTTTGCTTGCTTTATCCTGTG
	Reverse	CTGGAATGTGTTGCGTGGAT
18S	Forward	GTAACCCGTTGAACCCCATT
	Reverse	CCATCCAATCGGTAGTAGCG
U6	Forward	AACGCTTCACGAATTTGCGT
	Reverse	CTCGCTTCGGCAGCACA
miR-7-1-3p	RT	GTCGTATCCAGTGCAGGGTCCGAGGTATTCGCA CTGGATACGACTATGGC
	Forward	CGCGCAACAAATCACAGTCT
miR-7-5p	RT	GTCGTATCCAGTGCAGGGTCCGAGGTATTCGCA CTGGATACGACAACAAC
	Forward	CGCGTGGAAGACTAGTGATTTT
miR-24-3p	RT	GTCGTATCCAGTGCAGGGTCCGAGGTATTCGCA CTGGATACGACCTGTTC
	Forward	GCGTGGCTCAGTTCAGCAG
miR-30a-5p	RT	GTCGTATCCAGTGCAGGGTCCGAGGTATTCGCA CTGGATACGACCTTCCA
	Forward	CGCGTGTAAACATCCTCGAC
miR-30d-5p	RT	GTCGTATCCAGTGCAGGGTCCGAGGTATTCGCA CTGGATACGACCTTCCA
	Forward	GCGTGTAAACATCCCCGAC
miR-30e-5p	RT	GTCGTATCCAGTGCAGGGTCCGAGGTATTCGCA CTGGATACGACCTTCCA
	Forward	GCGCGTGTAAACATCCTTGAC
miR-30c-1-3p	RT	GTCGTATCCAGTGCAGGGTCCGAGGTATTCGCA CTGGATACGACGGAGTA
	Forward	GCGCTGGGAGAGGGTTGTT
miR-30c-2-3p	RT	GTCGTATCCAGTGCAGGGTCCGAGGTATTCGCA CTGGATACGACAGAGTA
	Forward	CGCTGGGAGAAGGCTGTT
miR-181b-3p	RT	GTCGTATCCAGTGCAGGGTCCGAGGTATTCGCA CTGGATACGACTTGCAT
	Forward	CGCGCTCACTGAACAATGA
miR-542-3p	RT	GTCGTATCCAGTGCAGGGTCCGAGGTATTCGCA CTGGATACGACTTTCAG
	Forward	GCGCGTGTGACAGATTGATAA
miR-550b-3p	RT	GTCGTATCCAGTGCAGGGTCCGAGGTATTCGCA

		CTGGATACGACCAGTGC
	Forward	GCGCGTCTTACTCCCTCAG
miR-942-5p	RT	GTCGTATCCAGTGCAGGGTCCGAGGTATTCGCA
		CTGGATACGACCACATG
	Forward	CGCGTCTTCTCTGTTTTGGC
miR-3921	RT	GTCGTATCCAGTGCAGGGTCCGAGGTATTCGCA
		CTGGATACGACACAAGG
	Forward	GCGCGTCTCTGAGTACCATATG
miR-4653-5p	RT	GTCGTATCCAGTGCAGGGTCCGAGGTATTCGCA
		CTGGATACGACGGTGTT
	Forward	CGCGTCTCTGAGCAAGGCTT
miR-7162-3p	RT	GTCGTATCCAGTGCAGGGTCCGAGGTATTCGCA
		CTGGATACGACGCTGCT
	Forward	GCGCGTCTGAGGTGGAAC
miRNA	Universal reverse	AGTGCAGGGTCCGAGGTATT

1.2.Table S2. ROC analysis of the diagnostic efficacy of the differentially biomarkers in PCa.

Biomarker	Sensitivity (%)	Specificity (%)	AUC (95% CI)	P-value
3 lncRNAs + circ_0057558 + circ_0062019	85.0	89.2	0.935 (0.880-0.991)	< 0.001
3 lncRNAs + ITGBL1+ TGM4 + KRT15 + HOXA7	93.8	92.7	0.968 (0.931-1.005)	< 0.001
ITGBL1 + circ_0062019 + RP11-2 33A14.1	93.3	92.3	0.957 (0.909-1.005)	< 0.001

1.3. Table S3. Target miRNAs analysis of LAMTOR5-AS1 by LncRNASNP2, DIANA, and miRDA score.

target miRNA	LncRNASNP2 score	DIANA score	miRDB score
hsa-miR-550b-3p	-	0.96	93
hsa-miR-942-5p	>=1	0.805	63
hsa-miR-542-3p	>=1	0.851	69
hsa-miR-181b-3p	<1	0.716	65
hsa-miR-7162-3p	<1	0.835	81
hsa-miR-3921	<1	0.782	71
hsa-miR-4653-5p	<1	0.848	71

2.1. Fig. S1. qRT-PCR analysis of the gene expression levels of lncRNAs in prostate cells and tumor tissue FNA samples. DelncRNAs in prostate cells (A-F), in the mixture PCa tumor tissue samples, compared to the adjacent normal tissues samples (G), and tumor tissue samples compared to BPH tissue samples (H-K).

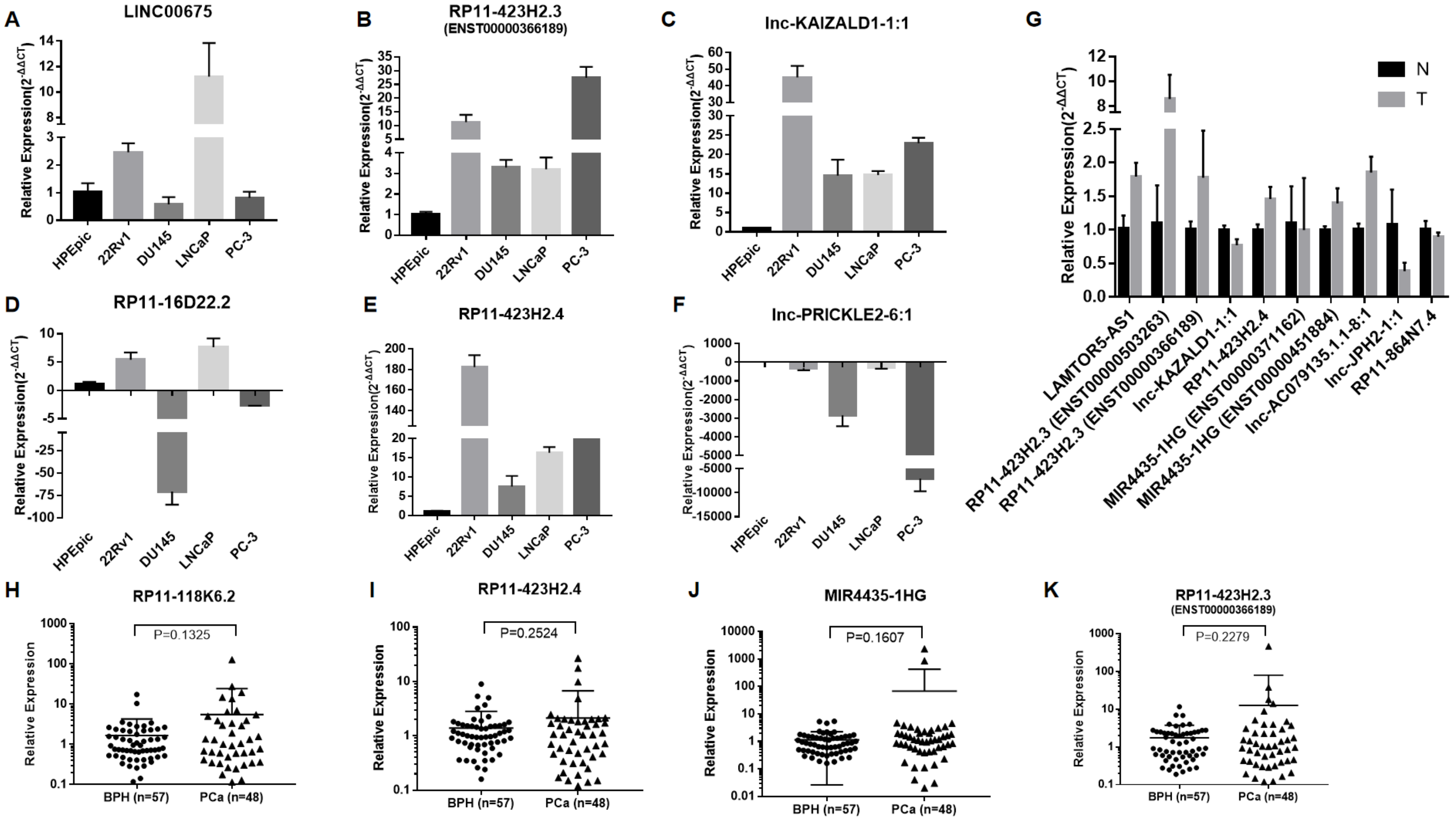

2.2. Fig. S2. qRT-PCR analysis of the lncRNA expression levels of LAMTOR5-AS1 in prostate tumor tissue FNA samples. GS6-7 (Gleason score 6-7: Less aggressive cancer); GS 8-10 (Gleason score 8-10: Aggressive cancer).

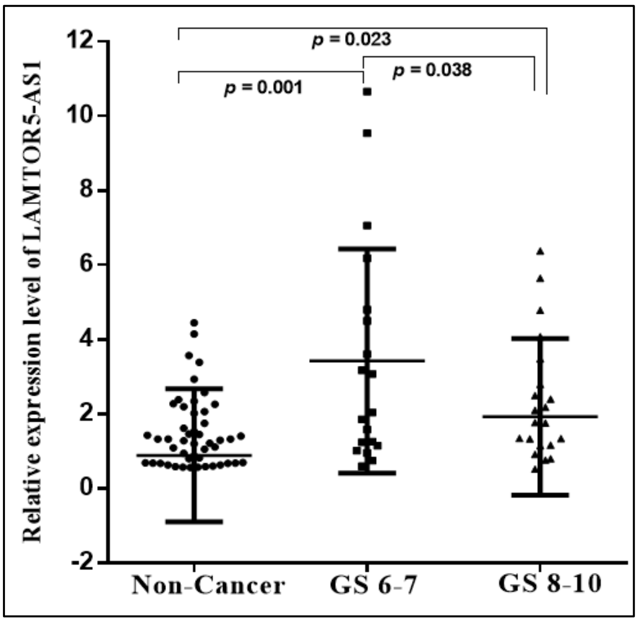

2.3. Fig. S3. ROC curve showing expression levels of three DelncRNAs (lncRNA RP11-33A14.1, RP11-423H2.3 and LAMTOR5-AS1), with two circRNAs (circ_0057558 and circ_0062019) or four DGEs (ITGBL1, TGM4, KRT15 and HOXA7) in PCa patients and BPH controls: the three lncRNAs and two circRNAs combination (A); the three lncRNAs and four DGEs combination (B); and the three lncRNAs and ITGBL1 and circ_0062019 combination (C). The ROC curves were analyzed using univariate (log-rank) analysis**.**

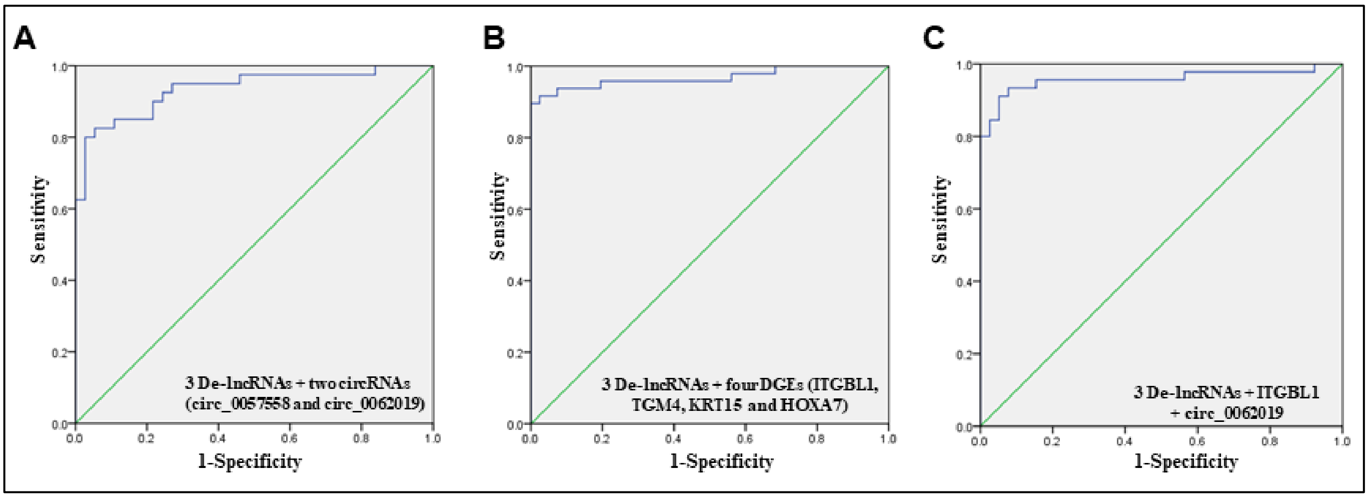

2.4. Fig. S4. Immunoblots analysis of RBP UPF1 and FUS expression in Prostate cancer PC3 and DU145 cells following knockdown of RP11-423H2.3 or LAMTOR5-AS1.

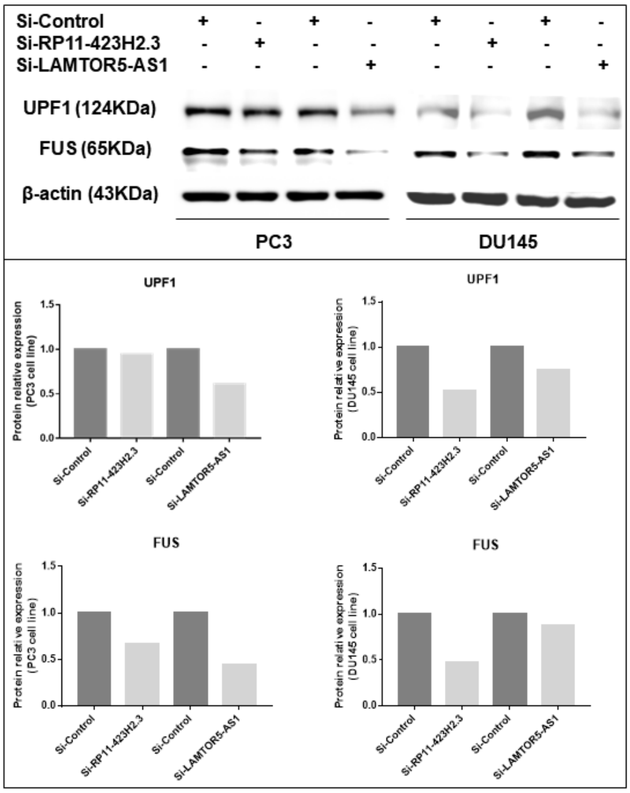

Supplement: Supplementary file 1 [file DataSheet_1.pdf]
